# “Do You Wanna Breathe or Eat?”: Parent Perspectives on Child Health Consequences of Food Insecurity, Trade-Offs, and Toxic Stress

**DOI:** 10.1007/s10995-015-1797-8

**Published:** 2015-07-09

**Authors:** Molly Knowles, Jenny Rabinowich, Stephanie Ettinger de Cuba, Diana Becker Cutts, Mariana Chilton

**Affiliations:** Drexel University School of Public Health, Philadelphia, PA 19104 USA; Las Mile Health, Zwedru, Liberia; Boston University School of Public Health, Boston, MA USA; Hennepin County Medical Center, Minneapolis, MN USA

**Keywords:** Food insecurity, Depression, Child development, Toxic stress

## Abstract

**Objectives:**

This study among 51 parents of young children under age four investigated how parents that report marginal, low and very low food security characterize how trade-offs associated with food insecurity affect parents’ mental health and child well-being.

**Methods:**

We carried out 51 semi-structured audio-recorded interviews after participants responded to a survey regarding food security status and maternal depressive symptoms. Each interview was transcribed. Through a content analysis, we coded “meaning units” in each manuscript and organized them by themes in ATLAS.ti. Among participants reporting both food insecurity and depressive symptoms, we identified three primary areas of concern: trade-offs, mental health, and child well-being.

**Results:**

Parents described how trade-offs associated with food insecurity have a profound relationship with their mental health and home environment that strongly affects young children. Descriptions of hardships include anxiety and depression related to overdue bills and shut-off notices, strains with housing costs, and safety. Parents described how their own frustration, anxiety, and depression related to economic hardship have a negative impact on their children’s physical health, and their social and emotional development.

**Conclusions:**

Parents in food insecure households recognize that trade-offs between food and other basic necessities are associated with their personal stress and poor mental health that, in turn, affects their children’s health and development. Partnerships between healthcare providers, policymakers, and parents are essential to successfully address and prevent the poor child health outcomes of toxic stress associated with food insecurity and poverty.

## Significance

*What is already known?* It is already known that adversity has damaging effects on child health and development, and that maternal/parental depression is a suspected mechanism.

*What this study adds?* This study offers a new perspective that adversity associated with household food insecurity is a form of toxic stress that is readily recognized by parents. It also demonstrates that parents have strong conviction that they can protect their children, yet admit their stress and anxiety make it difficult. Results suggest the need for public assistance programs to integrate behavioral health and comprehensive 2-generation approaches that help caregiver and child simultaneously.

## Introduction

In 2013, household food insecurity was reported by 20.9 % of US households with children under age 6, and by 34.4 % of female headed households [1]. Household food insecurity includes *low food security* (LFS), indicating reductions in quality, variety, and desirability of food without reductions in quantity, and *very low food security* (VLFS), which includes disrupted eating patterns and reductions in food intake in addition to low food security (Table 1). Although it is excluded from food insecurity estimates, marginal food security, meaning anxiety and occasional problems accessing adequate food, is also associated with negative health consequences like those reported for low and very low food security [2].

**Table 1:** United States Department of Agriculture Household Food Security Definitions

Household food security status	Definition
*Food secure*	
High food security	Households had no problems or anxiety about consistently accessing adequate food
Marginal food security	Households had problems at times, or anxiety about accessing adequate food, but the quality, variety, and quantity of their food intake were not substantially reduced
*Food insecure*	
Low food security	Households reduced the quality, variety, and desirability of their diets, but the quantity of food intake and normal eating patterns were not substantially disrupted
Very low food security	At times during the year, eating patterns of one or more household members were disrupted and food intake reduced because the household lacked money and other resources for food

Marginal food security and household food insecurity cannot be disentangled from trade-offs families make among other necessary expenses [3–7]. Trade-offs are perceived forced choices between paying for a variety of basic necessities because of financial limitations. Other related hardships can also precipitate such trade-offs. Research demonstrates that housing insecurity (overcrowding, or ≥2 moves in the previous year) and energy insecurity (having utilities cut off or using a cooking stove to heat one’s home) are associated with increased reports of food insecurity [4, 8, 9]. In addition to trade-offs of basic needs, food insecurity is associated with high rates of adult chronic disease and poor mental health [10–17], and poor physical health and greater developmental risk among young children [4, 18, 19].

Recently, the relationship between severe economic hardship and child development has been characterized as a form of “toxic stress.” [20, 21]. “Toxic stress” indicates overwhelming stress associated with economic deprivation and other forms of adversity, such as abuse, neglect, exposure to violence, and household instability, that can cause long-lasting physical and emotional damage [21]. There is growing evidence that food insecurity can and should be considered a form of toxic stress [22–26].

Despite research demonstrating damaging effects of toxic stress on child health and development, few studies have investigated these effects from the perspectives of parents. Parents are uniquely positioned to understand the toxic stress of food insecurity and its far-reaching consequences, and are important partners for clinicians, public health practitioners, and policymakers in addressing and preventing poor health and development in their children. Public health interventions meant to treat and prevent toxic stress are now prioritizing a two-generational approach and consist of coordinated multi-level efforts that include clinical, neighborhood, and community-wide interventions [27, 28]. Yet the perspectives and priorities of parents, and the enormous toll that stress of adversity takes on parents and in turn their children are rarely described nor incorporated in these calls for two-generation approaches. The stress of being behind on bills, skipping meals, going from house to house, escaping violence, and all the while trying not to pass the stress onto their children, needs to be explored and addressed in our intervention efforts. Our research with families reporting depressive symptoms and marginal, low and very low food security provides insight into the variety of ways that the stress of trade-offs between paying for utilities, housing, food, and healthcare negatively affect child health and development, and identifies potential avenues to reduce this stress.

## Data and Methods

Fifty-one parents of at least one child under age 4 were recruited from the ongoing Children’s HealthWatch (CHW) study in Philadelphia, PA (n = 21) and Minneapolis, MN (n = 30) between January and May 2010. Children’s Health Watch is an ongoing 5-site study in clinical settings investigating how public assistance programs and economic hardships, including food insecurity, affect the well-being of young children and their caregivers [18, 29]. After completing the CHW interview, interviewers asked parents to participate in a semi-structured interview regarding their experiences with food insecurity, coping strategies, health, and access to medical care, housing, employment, and child care. Participants were eligible for the additional qualitative portion of the interview if they were English-speaking, ≥18 years, born in the US or Puerto Rico, and reported marginal, low, or very low household food security according to the USDA Household Food Security Survey Module [30]. Interviews were conducted by trained interviewers in private rooms at a children’s hospital emergency department (Philadelphia) and an outpatient pediatric clinic (Minneapolis). Interviews were digitally recorded and lasted between 15 and 75 min. IRB approval for each site was obtained.

### Analysis

Interview transcriptions were managed with ATLAS.ti Version 6, a qualitative research software program. Transcriptions were analyzed in three stages. First, five research team members (two from Minneapolis; three from Philadelphia) carried out a content analysis by reviewing and coding “meaning units” or stories/experiences in the richest 10 transcripts to develop a scheme of 92 codes. These codes were categorized into four major themes: (1) hardship and stress, (2) coping and resources, (3) other household characteristics, and (4) social networks. Second, one senior researcher then coded all 51 interviews within ATLAS.ti. Third, after coding was completed, participant transcripts were categorized according to household food security status and depressive symptoms based on responses to the Kemper scale assessed during the CHW interview [31]. We then assessed the frequency and quality of coded quotations within each topic by food security and presence of depressive symptoms. Participants reporting both depressive symptoms and LFS or VLFS primarily expressed frustration and worry regarding “trade-offs,” the stress that trade-offs caused, and how they affected the health of their children (Table 2).

**Table 2:** Overview of qualitative themes overall and specific to this analysis

Theme (# coded meaning units)	Descriptions of groups of codes associated with the theme
*All top themes and brief description of associated codes*	
Hardship and stress	
Explicit stress (175)	Various stressful consequences of poverty, dissatisfactions with assistance programs, and the overall stress of life’s overwhelming challenges
Insufficient food (71)	Frequency and circumstances of being low on food or entirely running out of food, experiences with hunger
Lack of employment (54)	Circumstances of voluntary or involuntary unemployment, and specific impediments faced in finding employment
Inadequate assistance (105)	Inadequacy of support for basic needs of the amount of assistance that the respondents can obtain, and various obstacles experienced in obtaining assistance
Coping and resources	
Trade-offs (134)	A primary mechanism of managing food insecurity where there were perceived forced choices made between paying for basic necessities because of financial limitations
Social and financial coping strategies (144)	Mutual support from others; limiting both food intake and non-food expenditures
Personal coping strategies (237)	Personal strengths, such as acceptance of circumstances and various examples of personal resourcefulness
Emotional responses to hardship (143)	Sadness, frustration, resignation, worry and fear, and shame
Motivations and aspirations (134)	Supporting the ability to cope, primarily the universally pervasive finding in the data that concern for a child’s welfare, development, and safety dominate the parent’s behavior, choices and decisions
Household characteristics	
Housing (141)	Primarily describe unstable housing arrangements
Health and health care (161)	Impact of child ailments on resources and resource trade-offs, and the prevalence of depression that the respondents attribute to their extreme lack of resources
Social networks	
Social relationships (206)	Positive features of the social network, such as support from family and friends and the respondents’ appreciation and recognition of the value of social support, and negative features, primarily unsupportive or absent partners
*Selected themes and codes used for this analysis*	
Coping with trade-offs (589)	General trade-off; child before caregiver; food versus other expenses; nutrition compromise on quality
Mental health concerns (148)	Challenges just too much; depression, anxiety attacks; resignation; shame; social isolation; stress, sadness, feeling harassed; anger; worry, fear, anxiety
Positive/negative effects on child development/well-being (88)	Aware of or protective of child’s psychology; child ailments and health related needs; stress-based negative outcomes on kids

All names are pseudonyms. Ellipses with […] denote minor deletions that do not affect meaning; words or phrases within brackets [ ] denote replacement of names or phrases that are not the parent’s exact words, but still reflect the intended meaning.

## Results

### Participant Characteristics

As there were no significant differences in the participant characteristics between sites, demographics are presented for the entire sample. Seventy percent of parents were not married; 80 % had a high school degree or below; and 14 % were currently employed. Two-thirds of the families reported household LFS or VLFS, and over half of mothers reported depressive symptoms (Table 3).

**Table 3:** Characteristics of Sample

	N = 51	%
Total participants		
Minneapolis	31	59
Philadelphia	20	41
Gender		
Male	2	4
Female	49	96
Race/ethnicity^a^		
Black/African-American	39	76
Hispanic/Latino (any race)	6	12
White	3	6
Native American	1	2
More than one race	5	10
Marital status*		
Single^b^	23	45
Co-habiting	7	14
Married	10	20
Education*		
Some High School	19	38
Graduate High school/GED	21	42
Tech School/College Grad	10	20
Parent employment		
Unemployed	44	86
Employed	7	14
Household food security status		
Marginal food secure	12	24
Household low food secure	22	43
Household very low food secure	17	33
Child food security status		
Child food secure	30	59
Child low food secure	16	31
Child very low food secure	5	10
Maternal depressive symptoms^c^		
Yes	29	59
No	20	41

* Missing data

^a^Does not add up to 100 % because of rounding and participants reporting both race and ethnicity data

^b^Includes separated/divorced/widowed

^c^Depressive symptoms assessed for female caregivers only

Participants reporting LFS or VLFS (n = 39) compared to those reporting marginal food security (n = 12) were more likely to describe stress, insufficient food, inadequate assistance (such as inadequate amount of nutrition assistance, lack of access to housing assistance, and judgmental or unhelpful caseworkers), trade-offs, and social and financial coping strategies (such as limiting or skipping meals, asking friends/family for help, borrowing money, stealing money for food, and doing odd jobs). Similarly, participants reporting depressive symptoms (n = 29) were more likely to describe stress, lack of employment, and weak social networks than parents not reporting depressive symptoms (n = 20). Compared with parents who reported neither (n = 5), parents reporting household food insecurity *and* depressive symptoms (n = 23) were more likely to describe explicit stress, insufficient food, trade-offs, and emotional distress responses to hardship, including shame, resignation, worry and fear. Commonly described trade-offs included reducing quality of food to have sufficient quantity; parents reducing or eliminating their own meals so their children could eat; trading off food, housing, and energy payments with each other or with healthcare costs; and living in unsafe or unstable housing environments. Descriptions of child health problems included inadequate nutrition, asthma, sadness and depression, and behavioral problems. Below we describe parents’ assessment of the relationships between trade-offs, mental health concerns, and child well-being.

### Coping with Trade-Offs: “Do You Wanna Breathe or Eat?”

Participants described financial insecurity as a form of stress with deep impacts on their self-worth and capabilities, readily reporting economic hardship with frustration and resignation. In the words of Joanna, a mother from Minneapolis who reported VLFS:

That’s the hardest thing in life: to face reality. When you face reality then you goin’ somewhere. When you in denial, then you at a standstill. And I don’t want to be at a standstill. This is the way it is. We is poor and we is hungry.

All parents described not having enough money to cover basic family needs such as food, rent and utilities. Joan, a Philadelphia mother reporting LFS, described how she alternated bill payments, paying the rent 1 month, then the utility bill the next month, while she struggled to pay for food throughout. She lived with constant worry over having her utilities disconnected:

I have shut-off bills all over the place. […] I got it from the gas company. I got one from the water department. I got one from the [electricity] department, and I don’t have any phone in the house. Trust me: I got a shut-off bill from everybody. So it’s hard because I don’t like to live like that. I hate being scared. I hate getting up in the morning and hearing that drilling noise [from the utility company]. I’m like, “Oh my God, that’s for me! […] They’re gonna shut this off.”

These coexisting hardships and the imminent threat of having to make trade-offs are associated with fear and worry.

Trade-offs were often characterized as potentially compromising the parents’ and/or child’s health. Connie, a Philadelphia mother of four reporting LFS, provided an example of trading off between basic needs and the resulting impact on child health. She described how costs of healthcare and her children’s asthma treatments forced her to make trade-offs between buying food and maintaining good health. At times, either she or her children lacked medical coverage, despite the children’s eligibility for medical coverage. Recounting an emergency room visit for her son’s asthmatic crisis, Connie described not being allowed to leave the hospital unless she could prove she had a nebulizer. She felt forced to buy the nebulizer on a family member’s “maxed-out” credit card so she could be released. Here she describes the trade-off between health and food:

My kids are asthmatic and they need nebulizers and they need the albuterol. This is not something to play around with. […That’s why I said,] do you wanna breathe or eat? That’s the way it was. I don’t want my kids in the hospital. Because then I’m gonna get hit harder with medical bills and hospital bills and doctor bills.

These trade-offs characterize economic hardship and clearly link financial hardship, parental stress, and child health.

### Mental Health Concerns: “Feeling Blue in a Corner”

Despite reporting resignation to their reality, parents described multiple strategies for finding food when resources were low, including utilizing food pantries, borrowing money, going to homes of friends or family for meals, or sending children to relatives who would feed them. The majority of participants also described the oft-reported health trade-off when food was scarce: feeding their children but going hungry themselves [32]. Jill, a Minneapolis mother reporting VLFS, insisted she was “fine” as long as her daughter was “fine”:

Actually I’m doin’ fine. It’s just that sometimes I be on a budget and I can’t eat how much as I want. […] So it’s like I have to break it down, you know, ‘cause I’m so focused on her since she come first. So, as long as she’s taken care of I’m… I’m fine.

But this experience was not always consistent with parents’ descriptions of being “fine.” For some parents, the experience of not having enough money for food was associated not only with a physical experience of hunger, but also depression. Joanna described her physical and emotional experience this way:

It’s depressing because I’m okay with my kids going to sleep with a full stomach, or at least a satisfied stomach that they can go to sleep. But it’s uncomfortable for me to wake up and my stomach’s touching my back. …‘Cause now I’m upset ‘cause there’s nothing to eat here. [My kids are] looking at me like, “Okay, we ate yesterday, what about today?” So then I’m like, “Okay, now what do I do?” ‘Cause now we out of food […] We out of everything. It looks like a brand-new house through the cabinets, ‘cause there’s nothing there. So, it gets kind of depressing and frustrating all at once.

This type of depression and frustration were a constant backdrop among food insecure families, taxing mental resources already stretched by coping with crises intrinsic to economic hardship.

The crises went beyond not having money for food to include struggles with housing and exposure to violence. Several parents described feeling forced by economic constraints to live in unsafe environments in which they and their children were exposed to community and family violence, inadequate and insecure housing, and social isolation. Keisha, a Philadelphia mother of five, described concerns about her daughter’s development because she seemed overly attached to Keisha, always afraid to leave her mother’s side. Keisha attributed this to having lived in four homeless shelters in the previous several months, and to her daughter witnessing intimate partner violence Keisha experienced at the hands of her child’s father. Keisha described trying to hide her depression from her children:

So I’m always feeling blue in a corner thinking about what I’m going to do. Do you know what I’m saying? And you don’t want the kids to see you like this. So, you know, I go into the room and cry.

Depression, worry, fear and anxiety were common states described by most participants. Despite their best efforts to buffer their children, participants recognized their children were deeply affected by financial and emotional hardship.

### Trade-Offs and Mental Health Effects on Child Behavior: “Talking to Us in a Scream”

All participants, regardless of severity of food insecurity, expressed deep concern for their children’s safety and development. But among families reporting food insecurity, participants described difficulty protecting their children from depression and stress. Toni, a Minneapolis mother of two reporting VLFS and depressive symptoms, described her experiences of stress:

A normal day is like stress. Seriously. Like, most of the time, I be so stressed I just don’t wanna wake up. But then I know I have to wake up, because if I don’t wake up, then my kids is just gonna be running around with nobody. So I just wake up. Other than that, it’s just so stressful because my kids are so out of control.

Stress caused by lack of financial and social resources affected the behavior of the children as well as the parents. Several parents described how their stress was felt by their children, and affected their ability to be the kind of parent they hoped. Sheryl, a Philadelphia mother of two reporting LFS, had been living with her mother due to financial concerns but moved out because of violence between her mother and her mother’s boyfriend. Sheryl described being behind on rent and anxious about her children’s well-being. She explained how her children picked up on her emotional distress as she struggled to protect them from her anxiety:

When [my kids] really realize what’s really happenin’ is probably when I start cryin’—and they see me cryin’. That’s when they start cryin’. And then when I stop, that’s when they stop. They feel your feelings. When you cry or something, they feel it.

Her experience was echoed by Jim, a Minneapolis father of two reporting VLFS, who described how financial stress on the family is mirrored back to him and his wife through their child’s behavior:

[His mother] just got to the point where she doesn’t talk to my son anymore. She yells at him. So, in return, my son is talking to us in a scream. He doesn’t talk to us, he screams at us now. […] Somewhere along the line all this frustration, anxiety that all of us are feeling is just built up.

This exemplifies how financial stress affects parents, their ability to communicate effectively with their children, and their children’s behavior and well-being. Such experiences can spill over into other child behaviors, such as displays of aggression. Nancy, a Minneapolis mother of one reporting LFS, describes trying to contain her frustration this way:

When I need that break from [my son], there’s no one willing to take him. So it gets… like sometimes I get depressed. […] And I get angry with him because he’s violent with me. […] He’s really violent with me since I’ve been pregnant. […] We restrain him. We have to literally hold him and restrain him and it gets tiring.

As these parents describe, financial stress exacerbates the feedback loop between their own poor mental health and the behavior and mental health of their children.

## Discussion

These descriptions are vital windows through which to understand the experience of toxic stress created by economic hardship and food insecurity. The chronic, extreme stress of economic hardship, including food insecurity and basic needs trade-offs, is reflected in parent descriptions of experiences with depression, anxiety and fear. Parents described a direct connection between the trade-offs they are forced to make, their own emotional state, and that of their children. They also described how adversity associated with lack of access to food, lack of affordable housing, and exposure to violence are reflected in the behavior and well-being of their children.

The impacts of depression and food insecurity on child health and development have been described in quantitative studies, where maternal depression is on the pathway to poor child health among families reporting food insecurity [14, 33]. Yet most of these studies are done without parents’ narrative input, and without investigation of the contextual factors associated with toxic stress. Additionally, although this study did not intentionally seek to record exposure to violence, the casual references to violence in relationship to the poor mental health the parents described corroborate growing evidence that domestic violence, trauma and similar stressors are associated with food insecurity [15, 20, 34, 35]. While research has demonstrated the association between maternal depression, food insecurity, and poor child health and development [10, 11, 14, 29], this study shows that parents are not only aware of these connections, but are eager to find ways to protect their children from these different forms of stress.

Our results demonstrate that while parents are trying to buffer their children from food insecurity, resonating with other studies on the issue [32, 36], parents also recognize that they cannot truly shield their children in all domains of hardship, especially in the areas of stress and depression. Recent food security research with school-age children finds that parent and child reports of household food insecurity may differ within the same household; child reports indicate greater food hardship than parent reports. That work suggests that parents may try to minimize reports of the magnitude of their children’s food insecurity [37]. Almost all parents we interviewed expressed a strong desire to protect their children from food insecurity and the consequences of trade-offs, yet they recognized that their mental and physical state deeply affect their children’s current health, development and future. This is especially true of parents simultaneously reporting food insecurity and maternal depressive symptoms.

Parents’ perspectives must be included in efforts to address poor child health and development among food insecure families, and both clinical and policy interventions will have greater success if they capitalize on parents’ strengths, insights, and desire for a better life for their children. Educators and healthcare providers who recognize behavioral issues in children must consider how household food insecurity, financial stress and other material hardships, as well as attendant mental health of parents are affecting children’s health and development, and must seek ways to help parents address hardships. Ensuring that nutrition, housing, and energy assistance programs work in concert with each other may prevent parents from having to face trade-offs for basic needs, and incorporating mental health support into public assistance programs may help to alleviate the physical and mental health consequences of hardship for families and children. In addition, screening for food insecurity during health assessments would allow clinicians to identify toxic stressors and refer families to public assistance programs and mental health services.

As with all qualitative studies, this study is not meant to be representative of all food-insecure families. The recruitment and sampling technique may have biased results toward either greater reporting of stress associated with food insecurity, or potentially, under-reporting of the stress as those who experience even greater stress may not have been willing to participate. Low participation of fathers limits our understanding of experiences unique to male caregivers.

## Conclusion

Food insecurity, with its associated trade-offs and mental health consequences, creates a cluster of hardships corresponding to toxic stress for children and adults. Parent descriptions of families’ material hardships and harmful consequences of the resulting stress on their children suggest that parents recognize just how deeply adverse experiences can affect their children, even when they try hard to protect them.

Holistic family-focused policies that recognize the interconnectedness of parents’ and children’s needs are essential, and policies that support provision of housing assistance, consistent, affordable early learning opportunities for children, and material and mental health supports for parents seem all the more appropriate and urgent. Encouraging clinicians and early childhood educators to screen for hardship and refer for public assistance and behavioral health support could help alleviate not only material hardship, but also preventable and unnecessary stress and anxiety. Additionally, clinicians and policymakers should ensure that parents have more opportunities to describe their hardships directly to professionals and to partner with social service agencies to identify the solutions to address them.
